# How parents perceive screen viewing in their 5–6 year old child within the context of their own screen viewing time: a mixed-methods study

**DOI:** 10.1186/s12889-017-4394-5

**Published:** 2017-06-01

**Authors:** Janice L. Thompson, Simon J. Sebire, Joanna M. Kesten, Jesmond Zahra, Mark Edwards, Emma Solomon-Moore, Russell Jago

**Affiliations:** 1 School of Sport, Exercise & Rehabilitation Sciences, University of Birmingham, Edgbaston, Birmingham, B15 2TT UK; 2 Centre for Exercise, Nutrition & Health Sciences, School for Policy Studies, University of Bristol, 8 Priory Road, Bristol, BS8 1TZ UK; 3 The National Institute for Health Research Collaboration for Leadership in Applied Health Research and Care West (NIHR CLAHRC West), University Hospitals Bristol NHS Foundation Trust, Bristol, UK; 4 NIHR Health Protection Research Unit on Evaluation of Interventions, University of Bristol, Bristol, UK; 5 School of Social and Community Medicine, University of Bristol, Oakfield House Oakfield Grove, Bristol, BS8 2BN UK

**Keywords:** Screen-viewing, Children, Parents, Mixed-methods, Qualitative

## Abstract

**Background:**

Few studies have examined parental perceptions of their child’s screen-viewing (SV) within the context of parental SV time. This study qualitatively examined parents’ perceptions of their 5–6-year-old child’s SV within the context of their own quantitatively measured SV.

**Methods:**

A mixed-methods design employed semi-structured telephone interviews, demographic and SV questionnaires, objectively-measured physical activity and sedentary time. Deductive content analysis was used to explore parents’ perceptions of, and concerns about, their child’s SV, and management of their child’s SV. Comparisons were made between parent-child dyads reporting low (<2-h per day) versus high SV time.

**Results:**

Fifty-three parents were interviewed (94.3% mothers), with 52 interviews analysed. Fifteen parent-child dyads (28.8%) exceeded the 2-h SV threshold on both weekdays and weekend days; 5 parent-child dyads (9.6%) did not exceed this threshold. The remaining 32 dyads reported a combination of parent or child exceeding/not exceeding the SV threshold on either weekdays or weekend days. Three main themes distinguished the 15 parent-child dyads exceeding the SV threshold from the 5 dyads that did not: 1) parents’ personal SV-related views and behaviours; 2) the family SV environment; and 3) setting SV rules and limits. Parents in the dyads not exceeding the SV threshold prioritized and engaged with their children in non-SV behaviours for relaxation, set limits around their own and their child’s SV-related behaviours, and described an environment supportive of physical activity. Parents in the dyads exceeding the SV threshold were more likely to prioritise SV as a shared family activity, and described a less structured SV environment with minimal rule setting, influenced their child’s need for relaxation time.

**Conclusions:**

The majority of parents in this study who exceeded the SV threshold expressed minimal concern and a relaxed approach to managing SV for themselves and their child(ren), suggesting a need to raise awareness amongst these parents about the time they spend engaging in SV. Parents may understand their SV-related parenting practices more clearly if they are encouraged to examine their own SV behaviours. Designing interventions aimed to create environments that are less supportive of SV, with more structured approaches to SV parenting strategies are warranted.

## Background

A sedentary lifestyle is a risk factor for obesity and various chronic diseases independent of physical activity (PA) [1, 2]. Increased time spent engaging in screen viewing (SV) (e.g., watching television (TV), using computers, mobile phones, tablets, and playing video games) is associated with a higher risk of obesity, metabolic abnormalities, poor academic exam performance, and psychological difficulties in children [3–6]. SV behaviours track into adulthood [1, 3, 7], highlighting the need to identify strategies to reduce child SV.

The United Kingdom (UK) Department of Health guidelines for PA state that children should minimise the time spent sedentary for extended periods, while in 2013 the American Academy of Pediatrics recommended that parents should limit non-educational SV to no more than two hours per day [8, 9]. Although there are no national data from the UK examining time spent in non-educational SV for children aged 5 to 7 years, national data from this age group for sedentary time are available. These data indicate that sedentary time for boys and girls on weekdays (excluding time at school) is 2.8 and 2.7 h, respectively, and 3.8 and 3.9 h, respectively, on weekend days [10]. In 2015, the American Academy of Pediatrics published a news release in advance of forthcoming recommendations highlighting that research findings and policy statements lag behind the pace of digital media technologies, and that policies need to evolve accordingly [11]. They highlighted the ubiquity of SV which makes it challenging to set total time limits, and emphasised the need for parents to serve as SV role models.

Parents play a key role in the SV behaviours of young children. Factors associated with increased SV among young children include increasing age and the amount of time that parents spend SV, while higher socioeconomic position, parental rules, and perception of a safe neighbourhood are associated with less SV [12, 13]. Although some studies indicate that the number of household TVs, the presence of a TV in children’s bedrooms, and mother’s employment and educational status are associated with SV in young children [14, 15], the strength of these associations is unclear [12].

A systematic review of parental perceptions of healthy behaviours to prevent overweight and obesity in young children concluded that a major barrier to PA is their child’s preferences for SV; however, while some parents expressed a desire for alternative PAs that could occupy their children at home, others did not view SV (in particular, TV viewing) as detrimental due to its potential to be educational and provide opportunities for PA [16]. These findings suggest that interventions designed to reduce SV time in children may need to educate parents about the health risks of SV and how to change the family approach to SV, guide them on examining the content of their child’s SV, and may require parents to examine and change their own SV behaviours in addition to their child’s.

There is a growing body of literature examining SV in younger children (less than 7 years of age) and their parents. Recent evidence indicates that children aged 3–6 years are 3 to 5 times more likely to watch at least 2 h of TV per day if their parents watch more than 2 h per day [17, 18]. There are also a number of qualitative studies examining parents’ perceptions of the amount and quality of their young child(ren)'s SV behaviours [19–22]. A recent study from the UK reported that approximately 90% of mothers with children younger than 5 years reported that they were not aware of the guidelines for PA or sedentary behaviour, and found it difficult to define and quantify these behaviours in their children [19]. Focus groups with Canadian parents of 2.5–5 year olds indicated that most were not concerned about the amount of SV their child engaged in, but were concerned about the content of such SV [20]. Parents also identified a number of benefits of SV including its role as an educational tool, babysitter and bedtime coping device, and as a means to promote family bonding and PA (via active games). Similar findings were reported from focus groups conducted with parents of children aged 4–6 years from six European countries [21].

Although studies to date have quantified the SV of young children and their parents, and have explored parental perceptions about the amount and type of SV their child engages in, it is unclear how the SV time and behaviours of parents themselves may impact their SV-related parenting practices. To our knowledge, few studies have used a mixed-methods approach to explore parents’ perceptions of their child’s SV behaviours within the context of the parents’ own SV time. Previous studies have not quantitatively assessed SV time or behaviours of both young children and their parents, and this has prevented researchers from exploring parental perceptions of their young child’s SV within the context of parental SV time. The current mixed methods study aimed to address these gaps in the literature by quantitatively measuring the SV of both parents and their 5 to 6 year-old child, and examining parents’ qualitative perceptions of their child’s SV within the context of their own SV time.

## Methods

### Study design

This study utilized data from the B-Proact1v study, a large cross-sectional study conducted from 2012 to 2013 which examined factors associated with SV and moderate-to-vigorous intensity PA (MVPA) among 1267 5–6 year-old children and their parents, recruited from 57 primary schools in the greater Bristol area, UK. A mixed-methods design was employed in the present study that included the use of semi-structured telephone interviews, demographic and SV questionnaires, and objectively-measured (via accelerometer) MVPA and sedentary time. A detailed description of the sampling, recruitment, and data collection methods for the full B-Proact1v study is published elsewhere [18]. Ethical approval was granted by the University of Bristol School for Policy Studies Ethics Committee with written informed consent obtained from parents for both their own, and their child’s participation.

### Sample and recruitment

A total of 999 parents (78.5% of the sample) provided consent to be contacted about an interview. Our goal was to interview parents across the range of child MVPA levels and household deprivation found within the full B-Proact1v cohort. As such, the sample of 999 parents was stratified according to thirds of minutes per day the child spent in MVPA and level of deprivation at the household level (assessed via the Indices of Multiple Deprivation (IMD) using home postcode, a relative measure of deprivation in the UK) [23]. IMD indicates local area levels of deprivation across seven distinct domains of deprivation: Income; Employment; Health and Disability; Education Skills and Training; Barriers to Housing and Services; Living Environment; and Crime. This produced a 3-by-3 matrix of 9 categories of MVPA and deprivation (e.g., 1 = low MVPA and low deprivation, and 9 = high MVPA and high deprivation) [24]. For reporting purposes in the results, parents in the lowest one-third of deprivation for the sample were identified as Low Deprivation, those in the middle one-third of deprivation were identified as Mid Deprivation, and those in the highest one-third were identified as High Deprivation. We then purposively selected a sub-sample of 274 parents that broadly reflected the main B-Proact1v cohort with regard to child MVPA and level of household deprivation, and randomly selected from this sub-sample and invited parents to participate in an interview. Batches of participants were selected at five times over the duration of the study to stagger data collection, and to ensure a purposive sample reflecting MVPA and deprivation from within the sub-sample of 274 parents. Interviews were conducted until data saturation was reached within each of the 9 categories of MVPA/deprivation and across the full sample, resulting in 53 parents from separate families (19.3% of the purposively selected sub-sample) being interviewed. Participants received a £10 voucher as a token of appreciation for participating.

### Data collection

#### Objective measurement of MVPA and sedentary time

Parents and children were included in the analysis if they provided at least three days (two weekdays and one weekend day) of valid accelerometer (ActiGraph GT3X) data, which was defined as wear time of at least 500 min per day.

#### Qualitative interviews

Semi-structured telephone interviews were conducted by trained researchers. All interviews were audio-recorded and transcribed verbatim. The interview guide employed open-ended questions with additional probes to explore parents’ perceptions of their 5–6 year-old child’s SV. Parents were encouraged to discuss whether they had concerns about the amount of time and appropriateness of the content of their child’s SV, whether their child’s SV was influenced by others, and any strategies they used to manage their child’s SV.

#### SV questionnaire

All parents in the B-Proact1v study completed a questionnaire about their SV time (weekdays and weekend days), including separate questions about the use of TV, computers/laptops, games consoles, and smartphones. Parents also reported the same information (except for smartphone use) for their child. Response options for each item were: none; 1 min to 30 min; 31 min to 1 h; 1 to 2 h; 2 to 3 h; 3 to 4 h; 4 or more hours per day. Previous research has indicated that assessing TV viewing via parental response to a single question is moderately correlated (*r* = 0.60) with 10 day TV diaries among young children [25].

### Data analysis

To avoid influencing responses, parents were not informed of their own, or their child’s measured MVPA and sedentary time during the interview. These data were used as descriptive variables to place the SV of both the parent and child in context. As parents reported the SV of themselves and their child as categories of time (e.g., 2 to 3 h) for each discrete SV behaviour (e.g., TV, computer), this precluded the development of a combined SV variable and may have led to overestimation of SV time. To allow for the identification of parent-child dyads reporting relatively low versus relatively high SV time, the 2013 American Academy of Pediatrics-defined recommendation of <2 h of SV per day was used as a threshold [9]. Parent-child dyads in which the SV time for each of the reported SV behaviours was <2 h per day were defined as those who did not exceed the SV threshold. Parent-child dyads that engaged in ≥2 h of SV for at least one behaviour were defined as exceeding the SV threshold. Descriptive statistics (mean, standard deviation (SD), confidence intervals, frequencies) were calculated for all quantitative variables (IBM, SPSS version 21.0).

Transcribed interviews were uploaded to QSR N-Vivo 10 for analysis. Due to the corruption of one audio-file, analyses were conducted on 52 interviews (*n* = 49 mothers; 94.2%). Deductive content analysis was used to explore themes identified from existing literature: parents’ perceptions of their child’s SV; any concerns they have about their child’s SV; and, if/how they manage their child’s SV [26, 27]. Data analysis began with repeated reading of the transcripts by four trained researchers who were also involved in conducting the interviews. During the organisation phase, the themes were used to create a categorisation matrix into which data were coded via an iterative process using an unconstrained matrix, which allowed for the creation of new themes that arose from the data. The entire study team was involved in creating and finalising the categorisation matrix. Bi-weekly peer-debriefing discussions were held throughout the analysis process until consensus was reached on the name and content of each theme. For the purposes of the current study, analysis involved comparing responses between parents in the parent-child dyads who exceeded 2-h per day SV on both weekdays and weekend days, and parents in the dyads who did not exceed this threshold on either weekdays or weekend days.

## Results

### Participant characteristics

Of the 53 parents interviewed, 50 (94.3%) were mothers. As previously reported, 39.6% of the sample ranked in the lowest third of deprivation [24]. Duration of interviews ranged from 12 to 50 min (average = 26 min).

Table 1 includes the descriptive characteristics of the 52 parents and their children for whom data were provided and analysed. There were missing data on weekday SV for 3 parents and on weekend SV for 5 parents. There were no missing data on children’s SV. The majority of parents were white British, working part- or full-time, with two or more children living in the household, and two parents living in the home. The mean time spent in MVPA exceeded the current recommendations of 30 min/day for parents and 60 min/day for children. Parents spent an average of over 8.7 h/day in sedentary time, with children spending an average of almost 6.2 h per day in sedentary time. Almost three-quarters (73.1%) of parents reported engaging in ≥2-h per day for at least one SV behaviour during weekdays, with 86.5% engaging in ≥2-h per day SV for at least one behaviour during weekend days. Thirty-six parents (69.2%) reported engaging in ≥2-h per day for at least one SV behaviour during both weekdays and weekend days. Five parents (9.6%) reported not exceeding 2-h per day SV on either weekdays or weekend days.

**Table 1:** Demographic characteristics of the interview sample of parents (*N* = 52) and their children

	Parents		Children	
	Mean ± SD^a^	N (%)	Mean ± SD	N (%)
Age (yrs)	37.84 ± 5.98	---	6.16 ± 0.53	---
BMI (kg/m^2^) or BMI z-score^b^	26.00 ± 5.45	---	0.11 ± 1.02	---
MVPA (min/day)^c^	48.08 ± 20.36	---	66.67 ± 17.46	---
Sedentary Time (min/day)	526.74 ± 69.7		369.47 ± 49.04	
Index of multiple deprivation score for household	12.7 ± 10.6	---	---	---
Number of children in household:				
1	---	6 (11.5)	---	---
2	---	31 (59.6)	---	---
≥ 3	---	15 (28.8)	---	---
Ethnicity				
White British	---	45 (86.5)	---	---
Other	---	7 (13.5)	---	---
Employment				
Full-time	---	15 (28.8)	---	---
Part-time	---	25 (48.1)	---	---
Unemployed/Full-time parent	---	12 (23.1)	---	---
Parents in Household				
1	---	5 (9.6)	---	---
2	---	46 (88.5)	---	---
Not reported	---	1 (1.9)	---	---
Exceeded 2 h per day screen viewing threshold on weekdays	---	38 (73.1%)	---	18 (34.6%)
Exceeded 2 h per day screen viewing threshold on weekends	---	45 (86.5%)	---	38 (73.1%)

^a^ *SD* standard deviation

^b^ *BMI* Body mass index; value for children is BMI z-score

^c^ *MVPA* moderate-to-vigorous intensity physical activity

Eighteen parents (34.6%) reported that their child exceeded the 2-h SV threshold for at least one SV behaviour on weekdays, and 38 (73.1%) exceeding this amount for at least one behaviour on weekend days. There were 15 parent-child dyads (28.8%) that exceeded the 2-h SV threshold for at least one behaviour during both weekdays and weekend days, with 5 parent-child dyads (9.6%) that did not exceed this threshold for any behaviour on either weekdays or weekend days. The remaining 32 dyads were comprised of various combinations of exceeding/not exceeding the 2-h SV threshold on weekdays or weekend days. The most common pattern was for the parent to exceed the SV threshold on both weekdays and weekend days, with their child exceeding the threshold on weekend days only (*n* = 16, 30.8%). The second most common pattern was for both the parent and child to exceed the SV threshold on weekend days only (*n* = 7, 13.5%). During weekdays, 42.1% of parents and 27.7% of children who exceeded the SV threshold did so via television watching, while these values on weekends were 73.3% of parents and 71.1% of children.

### Parental perceptions and concerns about child’s SV

When analysing the interviews for all 52 parents, the majority (*n* = 47) expressed limited or no concern about their child’s SV. There was a clear distinction between the perceptions, concerns, and approaches used by parents to manage their child’s SV when comparing the parent-child dyads that exceeded the 2-h per day SV threshold on both weekdays and weekend days to the dyads that did not exceed the threshold on either weekdays or weekend days. Three main themes highlighting the distinctions between these dyads were identified: 1) Parent’s personal SV-related views and behaviours; 2) Family environment; and 3) Rules and setting limits.

#### Parent’s personal SV-related views and behaviours

Parents in the parent-child dyads that did not exceed the SV threshold described their preference for prioritizing and engaging with their children in non-SV behaviours for relaxation (e.g., reading), and highlighted how their own or their spouse’s upbringing influenced them to set limits around current SV-related behaviours for both themselves and their child(ren). Some parents also described negotiating around SV at difficult times of the day (e.g., at bedtime).

*“…it [SV] would never be the first port of call…first there would be kind of sitting down to read books or just sitting with me...I kind of use it [SV] in an extreme situation…My husband on the other hand, he was brought up with the telly and he has always said to me, ‘I don’t know why you don’t just sit them in front of it more’… but I don’t want it to be a habit that they grow up just using*.*”* (Interview 43 Mother, Weekday SV 30 min-2 h, Weekend SV 31 min-1 h, child gender female, child Weekday SV 1–30 min, child Weekend SV 31 min-1 h, Mid Deprivation).

*“… if she [her daughter] comes to me and says at ten to seven in the evening that she wants to start watching something…I have to assess what it is…I could say ‘well if you have a wash, brush your teeth, get ready for bed, then you could have that [TV] as your bedtime story.’ And sometimes they are not keen on that because we read together every night and it is so much part of the routine that I think they loathe to miss that, they really like the reading.”* (Interview 39 Mother, Weekday SV 31 min-1 h, Weekend SV 31 min-1 h, child gender female, child Weekday SV 1–30 min, child Weekend day SV 31 min-1 h, High Deprivation).

Parents in the parent-child dyads that exceeded the SV threshold were more likely to describe prioritizing SV as a shared family activity, and had views around setting SV limits that may or may not be consistent with the views of other family members.

*“…no [we don’t set limits around SV]…we do let her watch television whilst she’s eating her food…my family are very opinionated…they try to tell me what to let her watch on telly, but I don’t really listen to them…So I let her watch what I want to let her watch, and they don’t agree with it, but I’m the parent…”* (Interview 36 Mother, Weekday SV 2–2.5 h, Weekend SV 3.5–5.5 h, child gender female, child Weekday SV 2-3 h, child Weekend SV 2.5–4.5 h, High Deprivation).

*“Yes [down time]…that’s our telly time, we might sit down and watch a nice film or something on the telly that we are all interested in…sometimes we like watching the dancing programmes… but yeah recreational time is the telly, watching something on the telly or we might play a game…but that [playing a game] is very rare…”* (Interview 28 Mother, Weekday SV 2.5-4 h, Weekend SV 2.5-4 h, child gender male, child Weekday SV 2–3.5 h, child Weekend SV 2–3.5 h, High Deprivation).

#### Family environment

Parents in the parent-child dyads that did not exceed the SV threshold described an environment that was socially and physically busy, and one that supported their child and themselves to be physically active.

*“…literally every night she has got something [socially or physically active] on, and on a weekend we are always out and about. We go out on our bikes, you know I am quite active anyway…”* (Interview 24 Mother, Weekday SV 1.5-2 h, Weekend SV 1-2 h, child gender female, child Weekday SV 1–30 min, child Weekend SV 31 min-1 h, High Deprivation).

*“She seeks out activities…we live two kilometres, over a mile away from school, so mostly I car share but she [her child] is actually is very unhappy about it because she would rather walk, and if my husband is available in the morning he takes her, they walk to school.”* (Interview 39 Mother, Weekday SV 31 min-1 h, Weekend SV 31 min-1 h, child gender female, child Weekday SV 1–30 min, child Weekend day SV 31 min-1 h, High Deprivation).

In contrast, parents in the parent-child dyads that exceeded the SV threshold described an environment that was more relaxed and less structured, with many parents emphasising the busy lifestyles of their children and their need for relaxation time.

“…*she [her child] can put the TV on and watch it…I don’t really care, she doesn’t really sit there for long, she’ll put it on and it may be on all day but she’s not always watching it...she plays games, she’ll get herself something and get toys out and she’ll be playing, and as I say the TV will be on and she’s probably not even watching it because she’s playing*...” (Interview 3 Mother, Weekday SV >4 h, Weekend SV >4 h, child gender female, child Weekday SV 2-4 h, child Weekend SV >4 h, High Deprivation).

*“…I think there’s some parents that try to… make their children do too much…but I just know my child’s limits…I think some people believe that their children should be doing something every day after school, where I don’t. I believe that there should be certain days where we come home and, you know, just chill out at home*...” (Interview 16 Mother, Weekday SV 3-5 h, Weekend SV 3-5 h, child gender male, Weekday SV 2-4 h, child Weekend SV 3-5 h, High Deprivation).

#### Rules and limit setting

Parents in the dyads that did not exceed the SV threshold described setting clear rules limiting SV, controlling content, and in one case this included not having a TV.

*“For starters we don’t actually have a TV…we do have a DVD player and the children do watch some DVDs that we provide. At weekends the children play computer games, we do have Wii as well but they don’t play during the week…We have a rule that…there is no electronics until the homework is done...”* (Interview 39 Mother, Weekday SV 31 min-1 h, Weekend SV 31 min-1 h, child gender female, child Weekday SV 1–30 min, child Weekend SV 31 min-1 h, High Deprivation).

*“We do restrain what she watches on telly…she doesn’t have telly in her bedroom and any gadgets so that is really good…we are very, very tight with what she does…she will just go on my computer and she will just do like the school website and CBBC [online children’s games]…literally that is it.”* (Interview 24 Mother, Weekday SV 1.5-2 h, Weekend SV 1-2 h, child gender female, child Weekday SV 1–30 min, child Weekend SV 31 min-1 h, High Deprivation).

*“…we’re fairly regimented as to how much we let her see anyway, so it’s really keeping it [excessive SV time] at bay…if she’s watching something, and she’s watched enough, then we will say, ‘right that’s enough now, let’s go out, let’s go out in the garden and play instead’.”* (Interview 26 Father, Weekday SV 1-2 h, Weekend SV 1-2 h, child gender female, child Weekday SV 1-2 h, child Weekend SV 1-2 h, Mid Deprivation).

Parents in the parent-child dyads that exceeded the SV threshold described setting minimal rules around SV, with some parents describing high levels of educational SV as acceptable. Ten of these parents (66.7%) described the home environment around SV as very relaxed. Examples included accepting the reality of engaging in multiple SV behaviours, having the TV on in the background, and having multiple TVs in the home.

*“…I don’t [care] about the TV, if you want to watch it, you want to watch it…no, I don’t have to reduce it [child’s SV time]…so there’s no limit, she can just pick it up [her sister’s iPad] as and when [she likes]…we have five TVs in this house, so we all watch TV. I have it on in the kitchen when I’m cooking...we always have dinner at the table, but the TV will be on*.” (Interview 3 Mother, Weekday SV >4 h, Weekend SV >4 h, child gender female, Weekday SV 2-4 h, child Weekend SV >4 h, High Deprivation).

*“…he’ll watch a bit in the morning…and then he’ll watch a bit when he gets home from school, and he might have a bit sort of later on in the evening…But it’ll be a mixture of watching TV or playing something on the iPad or the laptop, or…we’ve just discovered a Wii thing…I think as parents, we…wouldn’t want him to be spending an awful lot of time doing it, but also in today’s society, he needs to know how to use it.”* (Interview 37 Mother, Weekday SV 2-3 h, Weekend SV 2-4 h, child gender male, child Weekday SV 2-3 h, child Weekend SV 2-4 h, Low Deprivation).

*“…I haven’t tried reducing the screen time, no…it’s one of them things that sometimes it’s on in the background, as opposed to them actually watching it.”* (Interview 21 Mother, Weekday SV >4 h, Weekend SV >4 h, child gender female, child Weekday SV 2.5-4 h, child Weekend SV >4 h, High Deprivation).

## Discussion

The data presented in this paper indicate that when parents’ concerns were examined in the context of their own and their child’s SV, there were clear differences in the perceptions, concerns, and approaches used by parents to manage their child’s SV between those parent-child dyads exceeding the 2-h per day SV threshold and those that did not. Those not exceeding the SV threshold expressed strong concerns about their child’s SV, and they described addressing their concerns by being more vigilant about SV and setting clear rules to limit SV engagement. These findings are consistent with previous research showing that TV viewing was substantially lower in children whose parents exhibited authoritative, less permissive parenting practices and applied relatively restrictive SV limits in the home [28, 29].

Similar results were reported from focus groups conducted with Australian parents and caregivers of preschool children [30]. In that study, SV was accepted by parents as an inevitable consequence of modern living, but many expressed concerns about SV and consciously restricted it to avoid over consumption. These findings are consistent with the American Academy of Pediatrics’s messages around setting limits and paying more attention to the quality of SV content than to the platform or time spent engaging in SV [11]. Thus, our findings illustrate the importance of measuring SV time and behaviours of both parents and children to: 1) gain a more complete understanding of the factors associated with young children and their parents who either exceed or fall within a threshold of 2 h SV per day; and 2) inform the development and delivery of interventions. Based on our results, interventions need to be appropriately targeted to parents and families to ensure they are not only aware of the detrimental effects of excessive non-educational SV, but also take into consideration the influence that parents’ own SV-related beliefs and behaviours can have on the SV environment of the home. Interventions that acknowledge that children and families are busy and need ‘down time,’ recognize that family SV time is important time together for some families, and encourage parents to use authoritative strategies in setting rules and limits rather than employing permissive approaches to managing SV could be effective in helping families strike a healthy balance between SV and non-SV activities.

In the present study, parents in the dyads that exceeded the SV threshold expressed fewer concerns with their child’s SV behaviours, and were less likely to report setting and enforcing SV rules. Previous qualitative studies also identified that parents expressed limited concern about their child’s SV, and that children who have more permissive parents are more likely to exceed the 2 h per day SV threshold [20, 21, 28, 29]. However, to our knowledge, there are no published studies to date that have quantified parental and child SV use and qualitatively explored parents’ views of their 5–6 year-old child’s SV in the context of their own and their child’s SV time.

One limitation of this study is that most parents who agreed to be interviewed were mothers, and as such we were unable to fully examine the views that fathers have about their child’s SV. Also, the majority of parents interviewed were living in two-parent homes, were White British, and were more physically active than the general UK population and therefore these findings may not be generalizable to families with young children across the UK [18]. Another limitation is that the time spent in various SV behaviours was self-reported and analysed as separate and distinct behaviours. This could have resulted in an over-estimation of SV. We were also unable to quantitatively assess the time spent engaging in educationally-based SV behaviours, and were not able to distinguish between work and home-based computer use amongst parents.

The main strength of this study is the use of a mixed-methods approach to examine parents’ perceptions of their child’s SV within the context of their child’s and their own SV time. This approach provided rich information about the SV of parents and their young child. To our knowledge this has not been done in other published studies. Other strengths are that we collected data on level of deprivation and objectively measured MVPA and sedentary time, allowing us to examine variations between these factors.

## Conclusions

There were differences in the perceptions, concerns, and approaches used by parents to manage their child’s SV between those parent-child dyads that did not exceed the 2-h per day SV threshold and those that did exceed this threshold. The lack of concern and relaxed approach to managing SV that was described by the majority of parents who exceeded the threshold suggests that there is a need to help some parents reconsider the balance between PA and SV in their family and help them to adopt more authoritative rather than permissive parenting strategies. Parents could also be guided in examining their own SV behaviours and considering how these might influence their SV-related parenting practices. These strategies could be incorporated into a family home use plan for all media as recommended by the American Academy of Pediatrics [9] to provide support for parents in better managing their own, and their child(ren)‘s SV.

## Abbreviations

BMI: Body mass index
IMD: Index of Multiple Deprivation
MVPA: Moderate-to-vigorous intensity physical activity
PA: Physical activity
SD: Standard deviation
SV: Screen-viewing
TV: Television viewing
UK: United Kingdom

## References

[1] Juonala M, Magnussen CG, Berenson GS, Venn A, Burns TL, Sabin MA (2011). Childhood adiposity, adult adiposity, and cardiovascular risk factors. N Engl J Med.

[2] Healy G, Wijndaele K, Dunstan DW, Shaw JE, Salmon J, Zimmet PZ (2008). Objectively measured sedentary time, physical activity, and metabolic risk. The Australian Diabetes, Obesity and Lifestyle Study (AusDiab). Diab Care.

[3] Jago R, Baranowski T, Baranowski JC, Thompson D, Greaves KA (2005). BMI from 3-6 y of age is predicted by TV viewing and physical activity. Int J Obes Relat Metab Disord.

[4] Ekelund U, Brage S, Froberg K, Harro M, Anderssen SA, Sardinha LB (2006). TV viewing and physical activity are independently associated with metabolic risk in children: the European Youth Heart Study. PLoS Med.

[5] Corder K, Atkin AJ, Bamber DJ, Brage S, Dunn VJ, Ekelund U (2015). Revising on the run or studying on the sofa: prospective associations between physical activity, sedentary behaviour, and exam results in British adolescents. Int J Behav Nutr Phys Activ..

[6] Page AS, Cooper AR, Griew P, Jago R (2010). Children’s screen viewing is related to psychological difficulties irrespective of physical activity. Pediatrics.

[7] Singh AS, Mulder C, Twisk JW, van Mechelen W, Chinapaw MJ (2008). Tracking of childhood overweight into adulthood: a systematic review of the literature. Obes Rev.

[8] Department of Health, Physical Activity, Health Improvement and Protection. Start Active, Stay Active: A report on physical activity from the four home countries’ Chief Medical Officers. 2011. https://www.gov.uk/government/publications/start-active-stay-active-a-report-on-physical-activity-from-the-four-home-countries-chief-medical-officers. Accessed 01 Aug 2016.

[9] American Academy of Pediatrics (2013). Policy Statement. Children, Adolescents, and the Media. Pediatrics.

[10] Scholes S, Mindell J, Craig R, Mindell J (2013). Physical activity in children. Health Survey for England 2012.

[11] Brown A, Shifrin DL, Hill DL (2015). Beyond ‘turn it off’: How to advise families on media use. AAP News.

[12] Hoyos Cillero I, Jago R (2010). Systematic review of correlates of screen-viewing among young children. Prev Med.

[13] Atkin AJ, Sharp SJ, Corder K, van Sluijis EM (2014). Prevalence and correlates of screen-time in youth: An international perspective. Am J Prev Med.

[14] Dennison BA, Erb TA, Jenkins PL (2002). Television viewing and television in bedroom associated with overweight risk among low-income preschool children. Pediatrics.

[15] Kourlaba G, Kondaki K, Liarigkovinos T, Manios Y (2009). Factors associated with television viewing time in toddlers and pre-schoolers in Greece: the GENESIS study. Aust J Public Health.

[16] Pocock M, Trivedi D, Wills W, Bunn F, Magnusson J (2010). Parental perceptions regarding healthy behaviours for preventing overweight and obesity in young children: a systematic review of qualitative studies. Obes Rev.

[17] Jago R, Sebire SJ, Edwards MJ, Thompson JL (2013). Parental TV viewing, parental self-efficacy, media equipment and TV viewing among preschool children. Eur J Pediatr.

[18] Jago R, Thompson JL, Sebire SJ, Wood L, Pool L, Zahra J (2014). Cross-sectional associations between the screen-time of parents and young children: differences by parent and child gender and day of the week. Int J Behav Nutr Phys Activ.

[19] Bentley GF, Jago R, Turner KM (2015). Mothers’ perceptions of the UK physical activity and sedentary behaviour guidelines for the early years (Start Active, Stay Active): a qualitative study. BMJ Open.

[20] He M, Irwin JD, Sangster Bouck M, Tucker P, Pollett GL (2005). Screen-viewing behaviors among preschoolers. Parents’ perceptions. Am J Prev Med.

[21] De Decker E, De Craemer M, De Bourdeaudhuij I, Wijndaele K, Duvinage K, Koletzko B (2012). Influencing factors of screen time in preschool children: an exploration of parents’ perceptions through focus groups in six European countries. Obes Rev.

[22] He M, Piché L, Beynon C, Harris S (2010). Screen-related sedentary behaviors: children’s and parents’ attitudes, motivation, and practices. J Nutr Educ Behav.

[23] Department for Communities and Local Government: Indices of Deprivation. http://data.gov.uk/dataset/index-of-multiple-deprivation. Accessed 01 Aug 2016.

[24] Kesten J, Jago R, Sebire SJ, Edwards MJ, Pool L, Zahra J (2015). Understanding the accuracy of parental perceptions of child physical activity: a mixed methods analysis. J Phys Activ Health.

[25] Anderson DR, Field DE, Collins PA, Pugzles Lorch E, Nathan JG (1985). Estimates of young children’s time with television: a methodological comparison of parent reports with time-lapse video home observation. Child Dev.

[26] Elo S, Kyngas H (2008). The qualitative content analysis process. J Adv Nurs.

[27] Hsieh H-F, Shannon SE (2005). Three approaches to qualitative content analysis. Qual Health Res.

[28] Jago R, Davison KK, Thompson JL, Page AS, Brockman R, Fox KR (2011). Parental sedentary restriction, maternal parenting style, and television viewing among 10-11-year olds. Pediatrics.

[29] Langer SL, Crain AL, Senso MM, Levy RL, Sherwood NE (2014). Predicting child physical activity and screen time: parental support for physical activity and general parenting styles. J Pediatr Psychol.

[30] Dwyer GM, Higgs J, Hardy LL, Baur LA (2008). What do parents and preschool staff tell us about young children’s physical activity: a qualitative study. Int J Behav Nutr Phys Activ.

